# Supplementary material for: Pathogen contingency loci and the evolution of host specificity: Simple sequence repeats mediate Bartonella adaptation to a wild rodent host
Source: PLoS Pathog. 2024 Sep 30;20(9):e1012591. doi: 10.1371/journal.ppat.1012591 (PMC11466379; doi:10.1371/journal.ppat.1012591)
Supplement: Attachment — Submitted filename: Bartonella EE Response to Reviewers 2024-09-04.docx [file ppat.1012591.s007.docx]

We thank the reviewers for their time and help improving our manuscript with their comments. Our point-by-point responses are below in blue text. Please note that all line numbers below refer to the “Revised Article with Changes Highlighted” document.

**Reviewer #1**

**Summary:** Ruth et al., investigated Bartonella adaptation in rodent through infecitons of Gerbillus andersoni or Gerbillus pyramidum, or alternating infection between the two species. Mutations in two mononucleotide simple sequence repeats (SSR) causing frame shifts (gene off) of Bartonella autotransporter TAA were identified after 20 passages in Gerbillus andersoni, but instead of in Gerbillus pyramidum. The authors also confirmed SSRs are conserved in other genes showing ON/OFF variation in Bartonella isolates that circulate in rodent communities in the Negev Desert, suggesting SSR-based contingency loci play roles in evolution of host specificity.

It is of great interesting to identified SSR mediated mutations of TAA which is host species specific tracking from almost 300 samples whole genome sequencing data. However, this manuscript falls short of defining a biologically-relevant role of the process they describe, as there is no convincing data linking their observations to a significant aspect of Bartonella adaptation.

**Major comment 1**: A major weakness of the study is that the biological relevance of TAA mutation has not been confirmed yet, although the authors found TAA inactivation showed higher bacterial loads in blood of *Gerbillus addersoni* only at day 5 post infection. Single time point difference cannot be defined as better fitness. Furthermore, the authors did not generate isogenic mutant strains (such as carrying a badE) to access a badE phenotype in rodent host.

**Reply:** We recognize that further research is needed to explore the exact role of mutations in *badE* in enhancing *Bartonella* fitness within *G. andersoni* hosts, and we have included this point in the manuscript (including with an added comment on lines 439─440). However, we believe that our findings strongly support the significant role of SSR mutations in *Bartonella* infections in this host species. Specifically, (i) the mutations in *badE* were observed only in lines passaged through *G. andersoni* hosts and not in lines passaged through *G. pyramidum* or during the *in vitro* evolution experiment and (ii) the *badE* mutant strain achieved a higher titer in red blood cells earlier in *G. andersoni* infections compared to the ancestral strain. It is also worth noting that, although we did not create isogenic mutant strains, the entire genome of the mutant strain used in the infection experiment was sequenced and found to be identical to that of the ancestor strain, except for the SSR mutation in *badE*.

**Major comment 2**: Bacterial autotransporters are macromolecular proteins that play important roles in self aggregation, or adhesion with host cell matrix. Expression of such big proteins is very energy consuming. Therefore, it is not surprising to identify OFF mutations in these proteins when these proteins are not required under certain environment (as observed that BadA is inactivated when serially passaged on agar plates). In this manuscript, badE was inactivated during infection in *Gerbillus andersoni* while stayed invariable under infection in *Gerbillus pyramidum*, indicating that badE is required for establishment of infection in *Gerbillus pyramidum*. However, no difference was identified from the capacity of badE mutant to infect *Gerbillus pyramidum* against ancestor. This contradiction should be well addressed in the manuscript.

**Reply:** We agree with the reviewer that one can expect to find OFF mutations in proteins involved in self-aggregation or adhesion with the host cell matrix when these proteins are not essential, such as under laboratory conditions. We also agree that the lines passaged solely through *G. pyramidum* may have not fixed this mutation because the function of the *badE* gene is essential in this host. We added this possible interpretation to the Discussion (lines 460─462). However, based on our findings, it remains unclear whether the lack of OFF mutations in *badE* in lines passaged solely through *G. pyramidum* indicates that this gene is crucial for infection establishment in this host or if it reflects a lower selection pressure from the immune response in this host, reducing the advantage of inactivating this gene. As further research is needed to distinguish between these possibilities, we prefer to avoid adding more speculative conclusions in the already comprehensive Discussion section.

**Minor comment 1**: Page9, line 182, “were presented”.

**Reply:** The typo was corrected

**Minor comment 2**: Page 21. Information of animals is too simple. *Gerbillus* is not a traditional lab animal. Are they adopted from wild environment or they are hand-fed? If they are captured from Negev desert, are they serum negative of *Bartonella* infection? It will be very important for the interpretation of the data.

**Reply:** We appreciate the reviewer’s comment and added a section titled “Rodent hosts” to the Methods. This paragraph describes the source of our rodent lab colony—progeny of wild rodents that have been born and raised in the laboratory for about six years under semi-natural conditions, their maintenance conditions, their age and reproduction status, and the fact that they have never been exposed to ectoparasites or any *Bartonella* species, nor have they received any drug treatment (lines 497─506).

**Reviewer #2**

**Summary:** In this manuscript the authors study within host adaptation of the pathogenic bacteria Bartonella, whose strains naturally infect different species of rodents in the Negev Desert.

Using experimental evolution within two different host species and whole genome sequencing of the bacteria, the authors aimed at testing if the mutation rates and the genomic basis of adaptive evolution depended on the host and on the history of host infections.

By performing sequential infections of a strain into rodents in the laboratory during 20 infection cycles, they find that mutations in single sequence repeats (SSRs) of an autotransporter adhesion locus (which they call gene badE) arise independently. This bonafide adaptive locus is common across passages in a specific rodent species and also in infections where the hosts alternate. The SSR mutations are not seen during in vitro passages in a mutation accumulation experiment performed previously, strongly suggesting that the cause of their emergence in the infection is selection within a specific host and not hypermutation.

Their evolution data suggests that SSRs in badE contributes to increased fitness of the strain in specific hosts, and this hypothesis was confirmed by further in vivo infections with the ancestor strain and with an evolved mutant clone.

Interestingly, the adaptive region identified in the laboratory experiments is polymorphic in nature, which suggests that the identified locus contributes to the adaptation of this bacteria to host shifts. Finally the authors put the results in a broader phylogenomic context.

**Minor comment 1**: “Pg 10 Fig 3 Would be nice to indicate males and females with different symbols.”

**Reply:** Given the existing variation across time points and between host species and bacterial strains, we believe that introducing another level of complexity to the figure could divert the readers’ attention from the key finding: the earlier infection of the mutant strain compared to the ancestral strain in *G. andersoni* rodents.

**Minor comment 2**:”Pg 17 Line 357-360. "Theoretical and empirical studies ....". Please had citations”

**Reply:** Following the reviewer’s suggestion, we added six references related to the various points discussed in this section (lines 379-383).

**Minor comment 3**: “Pg 27 Line 597 typo in “downsamled””

**Reply:** The typo was corrected.

**Reviewer #3**

**Summary:** Rodríguez‐Pastor et al. presented a study of the molecular evolution in parasites during the adaptation to alternative hosts. They selected and carefully justified a host-parasite system involving bacterial parasite Bartonella krasnovii and two rodent hosts Gerbillus andersoni and G. pyramidum, sampled from wild communities in the Negev Desert in Israel. This system is a close simulation of real-world host-parasite coevolution. In their main experimental evolution analysis, they involved a single colony isolate of parasite, and three host scenarios (either or both hosts), and 15 days by 20 infection cycles on each of 5 lines. This experimental setting has sufficient power to address the question of interest. They identified a total of 20 unique mutations across the experiment, and noted the recurrence of frameshift mutations in the mononucleotide simple sequence repeats (SSRs) of the what they named as badE adhesin genes. They investigated the biological roles and evolutionary patterns of these mutations, and proposed that these mutations confer evolutionary advantages during the infection of G. andersoni -- the harder host of the two. They found genetic evidence from wild Bartonella isolates of the same region that support this hypothesis.

Overall, this is a high-quality study of genetic and evolutionary mechanism underlying host specificity of parasites. It is thoughtful, comprehensive and logically sound, addressing an important biological question with considerable success. The experimental design and the model system will inspire future researchers. I did not identify any notable weakness in the methods and results. Therefore, I endorse the publication of this manuscript in PLOS Pathogens.

**Minor comment 1**: There is only one limitation that I wanted to note to the authors. The study does not involve flea as an essential vector during the transmission of *Bartonella* parasites among rodent hosts. The authors briefly admitted this in the discussion. I think it is reasonable to guess that the mutations of the badE gene may have detrimental effects during the flea stage, which may explain why naturally occurring mutations are not as dominating as in the experiment. I wouldn't suggest that the authors make significant modification of the manuscript, which is already sufficiently comprehensive, but I think some elaboration in the discussion will be useful.

**Reply:** We appreciate the reviewer's valuable suggestion. In response, we have used the potential cost of *badE* mutations to fleas as an example to highlight other aspects of the natural environment that may be significant but were not addressed in our study (lines 460−462).

**Minor comment 2**: Finally, I wanted to make a note on the term "parasite" that describes Bartonella. In my knowledge, which might be outdated, parasites usually refer to eukaryotes but not prokaryotes. In the rodent-flea-Bartonella system, flea is usually considered as the (ecto)parasite, whereas Bartonella is usually considered as the pathogen / symbiont. If my question is valid, it will make sense if the authors justified the choice of terms (but does not need to change the term).

**Reply:** We acknowledge that in parasitology, "parasites" usually refers to eukaryotes rather than prokaryotes. However, we employed the term "parasites" in a broader ecological and evolutionary sense, describing any organism that lives in or on a host while extracting resources at the host's expense. To clarify this terminology issue, we now use "parasites" only in this broader context and explain that it includes “pathogens”, while throughout the manuscript we specifically refer to *Bartonella* as “pathogens” as the reviewer suggests. The changes include substituting “pathogens” for “parasites” in the title of the article.
